# New Perspectives of Infections in Cardiovascular Disease

**DOI:** 10.2174/157340309788166679

**Published:** 2009-05

**Authors:** Ignatius W Fong

**Affiliations:** University of Toronto, Division of Infectious Diseases, St. Michaels’ Hospital, 4CC 179 Cardinal Carter Wing, 30 Bond St., Toronto, Ontario, M5B 1W8, Canada

## Abstract

Infections have been recognized as significant causes of cardiac diseases for many decades. Various microorganisms have been implicated in the etiology of these diseases involving all classes of microbial agents. All components of the heart structure can be affected by infectious agents, i.e. pericardium, myocardium, endocardium, valves, autonomic nervous system, and some evidence of coronary arteries. A new breed of infections have evolved over the past three decades involving cardiac implants and this group of cardiac infectious complications will likely continue to increase in the future, as more mechanical devices are implanted in the growing ageing population.

This article will review the progress made in the past decade on understanding the pathobiology of these infectious complications of the heart, through advances in genomics and proteomics, as well as potential novel approach for therapy.

An up-to-date, state-of-the-art review and controversies will be outlined for the following conditions: (i) perimyocarditis; (ii) infective endocarditis; (iii) cardiac device infections; (iv) coronary artery disease and potential role of infections.

## INTRODUCTION

Infectious agents have long been recognized as causes of diseases involving various components of the structure of the heart-- pericardium, muscle, endocardium, valves, autonomic nerves and more recently the vessels of the heart. The spectra of microorganisms causing diseases of the heart are very broad and include all classes of microbes-- i.e. viruses, bacteria (including myocoplasma, Chlamydia, Rickettsiae and mycobacteria), fungi and parasites.

The mechanisms by which microbes produce diseases of the heart varies with the specific entity, but include direct invasion with damage produced by inflammatory reaction mediated by cytokines, and chemokines; indirect damage by autoimmune mechanisms through molecular mimicry or reaction to released and exposed host antigens; and possibly through metabolic disturbances that predispose to atherogenesis; and the effects of circulating toxins as in diphtheria (see Table **1**).

Much of the advances in the past decade are related to refinements in our knowledge of the pathobiology through strides in molecular techniques such as advances in genomics, proteomics, and use of informatics. These advances in our understanding of the pathogenesis of diseases have opened new avenues to novel approaches to the diagnosis, treatment and prevention.

## PERIMYOCARDITIS

Classically, inflammation of the heart lining and muscle can present as primarily pericarditis, which is more benign and self-limited but often recurrent, or as myocarditis which can be asymptomatic and subclinical but on occasion can be fulminant with severe heart failure and death. The true incidence of perimyocarditis is unknown and difficult to ascertain as most cases are mild and subclinical. Autopsy series in unselected subjects have found prevalence of inflammation of the myocardium consistent with myocarditis in 1 to 9% of routine postmortem examinations [1]. Postmortem studies also found that subclinical myocarditis accounts for about 20% of sudden, unexpected deaths in children and young adults, including athletes and military recruits [1].

Limited studies have been performed on live patients with clinical suspicion of myocarditis or dilated cardiomyopathy (DCM), using endomyocardial biopsy specimens and the Dallas criteria, established in 1986 [2] with proposed revision in 1991 [3], for histological evaluation. Limitations of endomyocardial biopsies that may affect sensitivity include sampling error due to tiny tissue sample, and interobserver variation in interpretation of histology. These limitations could explain the low incidence of confirmed myocarditis (less than 10%) in trials of suspected clinical myocarditis [4].

Infectious agents are the leading cause of perimyocarditis and probably account for more than 60% of all recognized cases. Although in the clinical setting the etiology is mostly undefined, it is believed that the majority of “idiopathic perimyocarditis” are of viral cause. There are at least 30 different viruses, 24 bacterial agents, 6 fungi and 7 parasites listed as etiology of perimyocarditis [5]. Viral myocarditis usually involve diffuse inflammation of the myocardium, whereas bacterial and fungal organisms usually cause focal myocarditis, secondary to seeding from the blood stream or from direct extension of adjacent organs in immuno-suppression. Moreover, myopericarditis has been reported following smallpox vaccination in the US military forces [6].

## VIRAL MYOCARDITIS

Viruses are the commonest causes of myocarditis in North America and other developed countries, whereas in South and Central America *Trypansoma cruzi *(Chagas disease) is the leading cause. The main viruses implicated in myocarditis are coxsackie viruses, other enteroviruses, adenoviruses, influenza viruses, cytomegaloviruses, and human immunodeficiency virus. In a surveillance performed in England and Wales between 1990 and 1993, 368 cases of myocarditis and/or pericarditis were reported [7]. Viruses were reported to cause 69%, bacteria were responsible for 13%, mycoplasma for 9%, Chlamydia for 4% and *Mycobacterium tuberculosis* for 2%. When restricted to myocarditis alone viruses were the etiology in 66% and *Mycoplasma pneumoniae* in 13.5%. Coxsackie B virus has been found in most studies to be the commonest viral cause of myocarditis, and in the British study [7] it was most frequently associated with a mixed picture of perimyocarditis, whereas the influenza virus was associated with pericarditis or myocarditis alone.

Early studies in the nineteen sixties and nineteen seventies found that almost 52% of myocarditis occurs in children and young adults (<40 years), 14% in those aged 40 to 59 years, and only 5% in adults over 60 [8], reflecting age related susceptibility. Young children and particularly infants under 6 months of age, are extremely susceptible to enteroviral infections. Other factors that may contribute to susceptibility to myocarditis include sex, nutritional status, pregnancy and genetic immune-related mechanisms.

The clinical presentation of myocarditis may also differ with age, as young children more commonly present with a febrile, viral-like illness with gastrointestinal and sometimes meningeal symptoms with subsequent or concomitant cardiac manifestations. The acute form with severe heart failure and multi-organ dysfunction is more common in neonates and young children, whereas adults usually present with subacute smoldering disease and less commonly with acute myocarditis [9]. Proving the etiology of myocarditis is difficult as standard viral serological and culture methods are of low yield, and even molecular methods of endomyocardial – biopsy specimens using polymerase chain reaction (PCR) and in situ hybridization may be insensitive because of sampling error from a small tissue biopsy. For instance, viral genome has been identified in less than 20% of patients with DCM [1]. Analysis of large myocardial specimens from partial ventrilectomy in 26 patients with DCM have detected minus-strand enteroviral RNA indicative of active infection in 78% of patients [10]. The only viral sequences detected in this group of patients were from Coxsackie B viruses such as B3 and B4. In another study of acute myocarditis in children, with myocardial tissue samples obtained by different means (endomyocardial biopsy, autopsy or from explanted hearts), PCR methods detected adenovirus in 15, enteroviruses in 8, herpes simplex virus in 2 and cytomegalovirus (CMV) in one [11]. A more recent larger study in children and adults utilizing multiple (up to nine) endomyocardial biopsies or explanted heart samples, analyzed specimens from 773 patients with acute myocarditis (N=624) and DCM (N=149) from 20 centers in the U.S. [12]. Viral genome was amplified in 38% of the samples from patients with myocarditis and 20% of patients with DCM. Adenovirus was identified as the most common virus identified (59.4% of all virus detected), with enteroviruses being the second commonest viruses in 35.5%, in the myocardium of children and adults with myocarditis and DCM. Only adenovirus (N=18) and enterovirus (N=12) were detected in DCM. Inflammatory changes in the myocardium consistent with acute myocarditis were more prominent in enterovirus positive cases (79%), compared to adenovirus (40%) which were more often mild or borderline myocarditis by the “Dallas” criteria. This pattern was also seen in the myocardium of DCM, of the 11 patients with inflammatory infiltrates, 9 had detectable enterovirus and 2 had adenovirus. Dual infection was also found in 26 patient samples with myocarditis. In this study control myocardial samples rarely demonstrate viral genomes (1.4%) [12]. The frequency of adenovirus in the myocardium of patients with clinical or subclinical myocarditis may be related to the recent identification of a common receptor for Coxsackie B viruses and adenoviruses [13]. 

In a further recent study from Germany 245 consecutive patients with DCM underwent endomyocardial biopsies with PCR analysis for viral genomic sequences [14]. Viral genomes could be amplified in 67.4%, the commonest was parvovirus B19 (51.4%), human herpes virus-6 in 21.6%, enterovirus in 9.4% and adenovirus 1.6%, with 27.3% showing mixed viral genomes. Histology did not reveal active or borderline myocarditis in any of the samples. These data should be interpreted with caution and suggest that many of the viral genomes detected may represent non-specific findings (without causation) and possibly contamination from blood or other source. Moreover, this study did not include concomitant normal heart tissues for controls undergoing PCR analysis.

Another recent report has added further murkiness to our ranking of specific viruses in the etiology of severe myocarditis. In immunosuppressed patients and heart transplant recipients, CMV is a common cause of cardiac infection but is not considered important in the normal host. A recent report from Finland on myocardial autopsy samples of 40 patients with fatal myocarditis and 12 controls (accidental deaths), describe their findings of viral nuclei acids [15]. Viruses were detected in 43% with CMV accounting for 15 of the 17 (88%) positives, with enteroviruses in only 1 patient and no adenovirus detected. Only one of the patient was immunocompromised and in situ hybridization assays were positive for CMV DNA in cardiomyocytes of 80% of the CMV PCR-positive samples. The control samples contained no CMV nuclei acid. The importance or relevance of CMV in the myocardium is in question as to cause and effect of myocarditis. Previous studies of >1100 patients with active or borderline myocarditis detected CMV DNA by PCR in only 3% (mainly biopsy specimens) [12,16].

How can we resolve these issues of few studies reporting viral genomes of atypical agents (not known cardiomyotropic) in high proportion of cardiac samples from subjects with DCM or myocarditis? There are several possible explanations for these unexpected reports: i) spurious results from false positive PCR, a well known problem if extraordinary precautions are not taken to prevent contamination; ii) true positive PCR but contamination by blood, which may be carrying the virus (i.e. Parvovirus B19 commonly occurs in children with viraemia and the organism is tropic for erythrocytes); iii) detection of latent virus that resides permanently in the body after infection (all herpes viruses including CMV); iv) reactivation of latent herpes viruses (CMV, HHV-6, etc) without causing disease, as a result of impaired T-cell function which may occur with malnutrition and critical illness (as in severe heart failure); v) geographical differences which reflect differences in incidence or prevalence of different viruses; vi) variations of susceptibility of different populations; vii) variations in techniques used for PCR, as most studies used different techniques (often “home-brewed”) without national or international standardization and verification. Thus, these studies with unexpected results need to be verified by several other centers with examination of specimens involving larger sample sizes.

At present enteroviruses, especially Coxsackie B group, are the best well studied and proven group of viruses to cause significant clinical myocarditis, which has been re-duplicated in animal models. Coxsackieviruses are universally present as human pathogenic enteroviruses in the *Picornaviridae *family. There are two serogroups, A and B, with 24 known Coxsackie A serotypes causing mainly respiratory and enteric diseases, and 6 known Coxsackie B serotypes (1-6) associated with mild to severe diseases of the heart, meninges, brain, upper-respiratory tract, liver and possibly pancreas [17]. Although all 6 group B serotypes have been detected or isolated in patients with heart muscle disease, serotype CVB3 has been the most consistently reported as the predominant cause of myocarditis, and implicated in 20% - 40% of acute myocarditis or DCM [18].

The clinicopathalogical classification of viral myocarditis can be defined as: 1) fulminant myocarditis, with severe heart failure from left ventricular or biventricular dysfunctional (within 3 weeks after onset of viral infection), resulting in either death, emergency cardiac transplantation, or full recovery; 2) subacute myocarditis with moderate left ventricular dysfunction and less distinct symptoms that are considered predisposition for DCM; 3) chronic active myocarditis, with moderate left ventricular dysfunction and indistinct symptoms that precedes or suggest more restrictive muscle damage and active scar); 4) chronic persistent myocarditis, with normal left ventricular function, but may present with sudden death and persistence of inflammation is evident on biopsy, and viral genome may be detected [18-20].

In clinical practice many patients presenting with symptoms of acute pericarditis also have myocardial involvement, myopericarditis. In a recent prospective cohort study of 274 consecutive cases of acute viral or idiopathic pericarditis, myopericarditis was found in 40 (14.6%); as defined by raised cardiac enzymes or new onset of focal or diffuse depressed left ventricular function [21]. Arrhythmia was the best predictor of myopericarditis (odds ratio = 17.6), and by 12 months normalization of echocardiography and treadmill testing occurred in 98% of cases.

## PATHOGENESIS OF VIRAL MYOCARDITIS

Experimental murine models of myocarditis utilizing Coxsackie virus B3 (CVB3) have demonstrated three distinct immunovirological and pathological phases of the disease [18,22]. The virus binds to epithelial cells by the membrane protein decay accelerating factor (DAF) as the primary attachment protein, and the coxsackie virus and adenovirus receptor (CAR) as an internalization receptor [18]. The acute viraemic phase of the illness is characterized by virus replication in blood and infected tissues (especially myocardium, spleen and pancreas). In the first 3 to 4 days postinfection there is evidence of early cardiomyocyte injury with focal dying myofibers (from both necrosis and apoptosis) and micro-vesicular vaculation without significant inflammation [23]. Once within myocyte the CVB3 produces proteases, such as protease 2A, that have an important role in viral replication and can affect host proteins such as dystrophin [24]. Cleavage of dystrophin may have a role in release of the virus from myocyte since viral infection is increased in the absence of dystrophin. Infection of cardiomyocytes stimulate a potent innate immune response with release of cytokines interleukin-1α (1L-1α), 1L-1β, 1L-6, 1L-18, tumor necrosis factor –α (TNF-α), TNF-β, and interferon –γ (IFN- γ) which results in inflammatory cell reaction [18]. Appearance of neutralizing antibodies about day 4 postinfection may have a role in limiting viral replication. 

The subacute phase of the disease (days 5-14) is heralded by the release of progeny virus in the interstituim, which results in considerable increase in lymphocytic infiltration and marked increase in proinflammatory cytokines. Natural killer (NK) cells upregulated by 1L-2 then kill virus-infected cardiomyocytes to limit virus dissemination. However, long term persistence or over-activation of NK cells may result in further myocardial damage due to excessive release of cytotoxic molecules (i.e. perforin) [18].

Between 15 – 90 days postinfection the chronic phase of myocarditis usually begins with clearance of the virus from blood and peripheral tissues. However, viral RNA and capsid protein may still persist in the heart, spleen and lymph nodes in mouse and human [25,26]. Persistently infected cells are mainly localized within the chronic inflammatory areas, and continued viral replication may be responsible for more rapid progression of viral myocarditis to heart failure and DCM. In humans and murine models during the chronic stage of myocarditis the inflammation subsides with increased healing, interstitial fibrosis, tissue calcification, ventricular dilatation and cardiac hypertrophy development [27]. There is evidence of persistent expression of proinflammatory cytokine (TNF-α), regulatory cytokine 1L-18, and immunosuppressive/ fibrinogenic cytokine tumor growth factor–β in mouse model heart on days 28 and 98 postinfection, with chronic inflammation as part of the tissue healing process [28].

Despite out increased knowledge from studies of the murine models of viral myocarditis, the exact mechanisms of the pathogenesis still remains somewhat controversial. The three main mechanisms proposed for cardiac injury are: 1. excessive immune-mediated destruction of the tissue by immune cells targeting virus infected cardiomyocytes; 2. autoimmune-mediated destruction of cardiomyocytes by autoreactive immune cells and/or autoantibodies induced by molecular mimicry between viral and host antigenic epitopes; and 3. direct virus-induced cardiomyocyte injury by cleavage of cellular proteins caused by release of viral proteases, that result in impairment of host RNA transcription and protein translation [18]. Moreover, the virus itself may directly induce apoptosis of the cardiomyocytes. Currently, the evidence supports all three mechanisms as contributing to myocardial injury and viral myocarditis.

Unfortunately our greater understanding of the pathogenic mechanisms of viral myocarditis and DCM have not led to more effective specific therapy. Specific antiviral drugs for viral myocarditis are being evaluated and hopes for therapeutic success in some patients are now renewed, as there are experimental evidences that high viral replication is related to severity of disease and that even in the chronic phase persistence of virus is associated with pathological changes. Two potential antiviral agents with broad spectrum activity against enteroviruses, by blocking uncoating of the virus, are pleconaril and isoxazoles (WIN54954). Pleconaril binds the hydrophobic pocket in viral capsid protein 1, inhibiting enteroviral attachment and entry, is still an investigational drug which was available on compassionate case basis. A few clinical trials in young children with enterovirus infections (meningitis and respiratory infections) reported decreased severity and duration of symptoms with pleconaril [29]. WIN54954, also an experimental antiviral drug, reduces mortality in murine CVB-3 myocarditis [30].

There is also evidence that carvedilol, a nonselective beta-blocker with additional alphaI-adrenergic blocking and antioxidant properties (on the market for years), has been shown to be cardioprotective in experimental myocarditis [31]. Carvedilol markedly altered myocardial lesions and increased IFN-α and 1L-12 production on day 7 with reduction of myocardial virus replication. Moreover, carvedilol demonstrated anti-inflammatory effect and decreased toxic oxygen radicals. Selective beta-blocker such as metoprolol and a high selective alpha 1-adrenergic blocking agent (bunazosin) had no effects in this model [31]. Hence, prompt clinical trials with this marketed agent for moderate-severe viral myocarditis and even DCM are warranted.

Other potential novel agents that need further investigations (i.e. phase I pharmacology and toxicity studies) include a French maritime pine bark extract (pycnogenol) which in the murine model was found to decrease encephalomyocarditis virus replication and suppress expression of proinflammatory cytokines in the heart [32]. 

## CHAGAS MYOCARDITIS

*T.cruzi*, a protozoan parasite, is endemic in most Central and South American countries, where it is estimated that 8 to 10 million people are afflicted with Chagas disease [33]. With the increasing immigration from Latin America more cases of Chagas disease will be recognized in the United States, where it is estimated 100,000 infected persons reside, mostly acquired in the endemic areas [34]. Transmission of the disease occurs predominantly in the poor rural areas of Latin American with the infected triatomine bugs, mainly in childhood. However, transmission also occurs through blood transfusion (reported in US as well), organ transplantation, vertically from mother to infant, and rarely from ingestion of contaminated food or drink [34-36]. Rare cases of autochthonous transmission have been reported in the US [37,38], and *T.cruzi* infected animals and vectors are found in many parts of the US [39,40]. The US food and Drug Administration approved a Chagas disease screening assay for donated blood at the end of 2006 [41] and by the fall of 2007 193 donated blood were confirmed positive [33].

## PATHOGENESIS OF CHAGAS DISEASE

Nearly 18 million people in Latin American are infected with *T.cruzi* but only one third progress to chronic Chagas cardiomyopathy (CCC) while the others are considered asymptomatic. The asymptomatic individuals are mainly responsible for blood and organ transmission. The acute phase of Chagas disease is often subclinical and remain unrecognized but occasionally patients can present with acute myocarditis. The majority of infected patients remain in the latent phase of disease for 10 to 30 years or even for life, and clinical manifestation (cardiac and digestive forms) occur at 2 -3 % every year.

The chronic cardiomyopathy of Chagas has been considered a paradigm of infection-induced autoimmune disease [42], with chronic inflammation and advanced cardiac pathology but scarce parasites. Antigen-specific and antigen –non-specific mechanisms by which *T.cruzi *infection activate T and B cells, leading to autoimmunity have been described [42]. Various *T.cruzi *antigens cross react with host antigens at the B and T cell level and molecular mimicry has been considered as the mechanism leading to autoimmunity and pathology of CCC. Immunization of these antigens (B13, cruzipain and Cha) and / or passive transfer of autoreactive T lymphocytes in mice lead to similar disease as found in humans with Chagas disease [42]. 

Human CCC is an inflammatory-dilated cardiomyopathy with a 2 : 1 predominance of CD8 + cells in relation to CD4 + T cells, with a Th1-type cytokine profile, probably secondary to local production of 1L-7 and 1L-15 [43]. IFN- γ appears to be the crucial element for parasite control in a murine model, as its absence determined a drastic increase in parasitaemia, tissue parasitism, leucocyte infiltration of the heart and mortality [44]. There is still debate on the issue of direct damage caused by the immune response to persistent parasites rather than autoimmunity. However, the two mechanisms may not be mutually exclusive of each other. In A/J mice infection with *T.cruzi* leads to the development of severe inflammation, fibrosis, and parasitosis in the heart accompanied by vigorous cardiac myosin-specific delayed type hypersensitivity (DTH) and antibody production at 21 days postinfection [45]. Treatment of infected mice with specific antiparasite agent (benznidazole) eliminated mortality and decreased disease severity, as well as reduced cardiac myosin-specific DTH and antibody production. Reinfection of mice with *T.cruzi *or immunization with myosin led to redevelopment of myosin-specific autoimmune response and inflammation. Thus, a direct link between the levels of *T. cruzi* and the presence of autoimmunity suggest that parasite elimination may reduce or eliminate autoimmunity in the chronic phase of infection [45].

Chronic Chagas cardiomyopathy is characterized by dilated cardiomyopathy complicated by frequent and complex ventricular arrhythmias and conduction defects. In patients neurotransmitter receptor autoantibodies (anti-M2 cholinergic autoantibodies) may contribute to the pathogenesis of Chaga’s disease dysautomia but not to ventricular dysfunction [46]. In acutely infected rats autonomic nerve endings are damaged during *T.cruzi*-induced myocarditis, but gradual recovery occurs after the acute phase [47]. In chronic infection of mice the hearts demonstrate intense inflammation and fibrosis, and decrease beta adrenergic and increased muscarinic receptor densities than normal controls [48].

## CLINICAL ASPECTS OF CHAGAS DISEASE

Most patients with acute infection of *T.cruzi* are unrecognized or develop mild, nonspecific febrile illness. Severe acute myocarditis or meningoenecphalitis are rarely detected [33]. The acute phase last about 4 to 8 weeks, and then enter the chronic latent phase without symptoms (indeterminate Chagas) for life (70 to 80%); and a minority (20 to 30%) slowly progress over the years to CCC. Familial aggregation of CCC and areas of endemicity indicates genetic predisposition for cardiomyopathy. Studies have implicated monocyte chemoattractant protein (MCP) – 1 gene polymorphism [49], HLA class II DRB 1 polymorphism [50], BAT1 (a putative anti-inflammatory gene) variants [51], and IL-1 gene cluster polymorphisms [52], as predisposition for Chagas cardiomyopathy.

The earliest manifestations of CCC are usually conduction system abnormalities (right bundle branch block or left anterior fascicular block), and segmentel left ventricular wall motion abnormalities; followed later by complex ventricular extrasystoles, ventricular tachycardia, sinus bradycardia, high degree heart block, thrombo-emboli from the heart, and progressive dilated cardiomyopathy with congestive heart failure [33]. There are four separate but somewhat similar classification schemes to guide presence and severity of Chagas cardiomyopathy, including the American Heart Association staging [33].

Diagnosis of the acute phase of Chagas disease can be made by peripheral blood smear from anticoagulated blood or buffy coat, as the level of parasitemia is high and trypomastigotes can be detected by microscopy. The parasitemia decreases within 90 days of infection spontaneously, and is undetectable by microscopy in the chronic phase. Diagnosis of chronic Chagas disease is made by serology, most often by enzyme linked immunosorbent assay (ELISA) and immunofluorescent antibody test (IFA), and two tests based on different antigens or technique are used in parallel to increase the accuracy of diagnosis [33]. A third assay maybe required for discordant results. PCR-based methods are more sensitive in the acute phase and variable in the chronic phase, but is mostly used as a research tool or for examination of cord blood. All children of infected mothers should be tested serologically at 9 to 12 months.

## TREATMENT OF CHAGAS DISEASE

Benznidazole and nifurtimox are the only proven antiparaitic drugs for Chagas disease, both can be obtained in the US from CDC under investigational protocols. Benznidazole is the preferred agent as it is better tolerated and is given orally (5-7mg/kg per day) in 2 divided doses for 60 days, alternatively nifurtimox (8-10mg/kg per day) in 3 divided doses for 90 days [33]. The major side effect of benznidazole are rashes (30%) with photosensitization, and reversible dose-dependent peripheral neuropathy (30%) late in the course, gastrointestinal symptoms and rarely bone marrow suppression. Nifurtimox adverse effects are more frequent and include gastrointestinal complaints in 30-70% of patients, central nervous system (CNS) toxicity (irritability, insomnia, disorientation and less commonly tremors and peripheral neuritis), concurrent alcohol increases the risk of adverse effects and should be avoided. Both drugs are mutagenic and should be avoided in pregnancy.

Although drug therapy is recommended for all cases of acute and congenital infection, reactivated infection (as seen with immunosuppression and acquired immunodeficiency syndrome), and in children < 18 years with chronic latent (indeterminate) infection; generally antitrypanosomal therapy should be offered to adults age 19 – 50 years without advanced cardiomyopathy. Clinical trials in acute Chagas disease with antiparasitic drugs shows parasitological cure in 60% to 85% of patients, reduction of severity and duration of symptoms; and more than 90% parasitilogical cure of congenital infection treated within the first year of life [33].

In the chronic phase of Chagas disease (asymptomatic) 2 randomized, controlled trials of benznidazole in children (6-12 years) showed clearance of infection in about 60% (seroreversion 3-4 years post-treatment, or reduction in xenodiagnosis) [52,53]. Recently, a nonrandomized trial in adults with indeterminate and chronic Chagas disease without heart failure demonstrated that benznidazole (5mg/kg/day) for 30 days, compared to no treatment, was associated with reduced progession of heart disease over 17 years, 12/283 (4%) *vs*. 40/283 (14%) adjusted hazard ratio, 0.24, p=0.002 [54]. A RCT is currently underway to assess the benefit of benznidazole in patients with mild to moderate Chagas cardiomyopathy [55]. 

There is currently no good evidence of the value of specific antiparasitic drugs for CCC and many patients are treated symptomatically to control heart failure, arrhythmias and with anticoagulation to prevent thrombo-emboli based on the individual clinical manifestation. The five year mortality for CCC with cardiac dysfunction is above 50% [56], and risk score for predicting death in Chagas cardiomyopathy has been valilated. Six independent prognostic factors identified with point score are: 1) New York Heart association class III-IV (5 points); 2) evidence of cardiomegaly (5 points); 3) left ventricular dysfunction on echocardiography (3 points); 4) nonsustained-ventricular tachycardia on 24 hour Holter monitoring (3 points); 5) low QRS voltage (2 points); and 6) male sex (2 points) [57]. For low risk (0 to 6 points) the 10 year mortality was 10%, moderate risk (7-11 points) was 44%, and high risk (12 to 20 points) was 84%. For patients with high risk planned cardiac transplantation may be the best option for suitable candidates if available. Automatic implantable cardioverter-defibrillators for those with malignant ventricular arrhythmia and recovery from cardiac arrest should be considered [58]. Routine use of spiranolactone to block aldoterone is probably warranted (as in other dilated cardiomyopathies), as in the hamster model spiranolactone attenuated the myocardial remodeling in Chagas cardiomyopathy, reduced inflammation and mortality during the chronic phase [59].

## PROGRESS IN INFECTIVE ENDOCARDITIS

Infective endocarditis (IE), an uncommon condition, despite improvements in diagnostic techniques, surgical management and antibiotic choices, still carries a high morbidity and mortality. Although the incidence have not changed significantly over the past 3 decades, there have been changing patterns in the type of microorganisms and underlying risk factors [60]. Much of the changing patterns of IE is due to improvement in social and economic conditions in developed countries, longer life scan and invasive methods of management of several diseases, which is a price we pay for medical advances.

Rheumatic heart diseases is a rare predisposion for IE in developed countries for several decades and has been supplanted by degenerative mitral and aortic valvular disease predisposing to IE, which increases with age and with greater predominance in males [60]. However, rheumatic carditis still remain common in developing countries. Recent prospective observational studies in Europe found that 47% of patients with IE had no known previous heart disease [61]. This survey conducted in France in 1999 [61] reported that prosthetic-valve IE (PVE) was 16% of all cases, but more recent European data collected in 2001 indicated that PVE was implicated in 26% of IE with 74% affecting native valves [62].

Intravenous drug abuses (IVDA) have the highest incidence of IE of 1-5% / year [63], followed by patients with prosthetic-valves (1-3% after one year, then 0.3 – 0.6% per patient year) [64], and in the general population the median incidence is 3.6/100,000 persons/year [60]. Nosocomial infection account for up to 22% of IE with mortality greater than 50%, primarily related to long term central catheters and surgical procedures (for hemodialysis), with < 50% having underlying valvular disease [65]. The primary pathogens being staphylococci and enterococci with mortality above 50%.

The microbiology of IE have not changed dramatically over the past decade, S*taphylococcus aureus* is now the single commonest organism for native-valve IE (about 34%) and PVE (23-30%), followed by oral streptococci (i.e. *S.viridans*) 14-17%, group D streptococci and enterococci for native valve IE, and coagulase negative staphylococci (CoNS) in PVE (about 17%) [61,62,66].

## PATHOGENESIS OF IE AND CARDIAC DEVICE INFECTION

The understanding of the adherence of bacteria to damaged or mechanical valves have improved in the past decade or more. The major pathogens of IE such as S*taphylococcus *spp., *Streptococcus* spp., and enterococci (accounting for more than 80% of IE) have the greatest ability to adhere to damaged valves [64]. Moreover, transient bacteremia with these organisms commonly occur with normal daily activities. Damaged endothelium and prosthesis results in stimulation of tissue factor and other procoagulants that results in deposits of coagulum on the valves. The coagulum contain large quantities of fibrin-fibrinogen, fibronectin, plasma proteins and platelet protein. Pathogens associated with IE bind to these proteins, causing colonization and proliferation following transient bacteremia. In experimental IE *S.aureus* relies on sequential fibrinogen-binding (for valve colonization) and fibronectin-binding (for endothelial invasion) [67]. In animals, fibrinogen-binding (but not fibronectin – binding) was associated with induction of endocarditis, but both fibrinogen-binding and fibrinonectin-binding were associated with disease severity. In cultured cell lines fibrinogen- and fibronectin binding act synergistically to cause invasion of cells [67]. Adherence of *S.aureus *to prosthetic valves and other intracardiac devices is also mediated by microbial surface proteins interaction with host-tissue ligands, such as FNbp A and B which bind to fibronectin; clumping factor which binds to fibrinogen; and collagen adhesin which binds to collagen [68,69].

The mechanisms of coagulase negative staphylococci (CoNS) infection of cardiac prostheses, including pacemaker/defibrillator leads are somewhat different. *Staphylococcus epidermidis* achieve attachment to surfaces of devices mediated by nonspecific factors (such as surface tension, hydrophobicity, and electrostatic forces) or by specific adhesins (proteinaceous autolysin encoded by the alt E gene and the capsular polysaccharide adhesin, likely encoded by the ica operon) [70,71]. The accumulative phase during which the bacteria adhere to each other to form a biofilm, is mediated by the polysaccharide intercellular adhesin. Significant advances have been made in the past decade in understanding the pathobiology of biofilm formation.

Biofilm is universal for all device related infections including those of the heart, and the vegetations of both native-valve IE and PVE consist of biofilm colonies [72]. By understanding the microbiology of biofilms may lead to novel therapeutic advances and prevention of IE and cardiac device infections. It certainly explains the need for prolonged, intravenous high dose antibioties to achieve cure of IE and the universal necessity for removal of infected cardiac devices, except for some cases of PVE. A possible explanation for our ability to cure many PVE without valve replacement, whereas all infected pacemaker and defibrillator etc needs to be removed, maybe that the biofilm colonies are restricted to the vegetations in PVE but are more extensive on the infected surfaces of other cardiac devices.

Biofilm is made up of a complex sessile community of microbial cells that is irreversibly attached to a substratum and to each other, embedded in a matrix of extracellular poylmeric substances (slime), and are more metabolically active than free floating (planktonic) microorganisms [72]. There are several features of biofilm infection that makes medical therapy difficult and usually prerequisite removal of the foreign body to attain cure. The mechanism of biofilm resistance to medical treatment involves local defect in both host’s humoral and cellular defences. There is decreased opsonic antibody response to the biofilm cell envelope proteins, impaired phagocytosis and killing by neutrophils [73]. Furthermore, extra-cellular slime substance from *S.epidermidis* can also inhibit lymphocyte activity and impair cell mediated function [74].

Moreover, biofilm organisms are inherently resistant to antibiotics, disinfectants or germicides compared to planktonic bacteria. The concentration of antibiotics required to kill biofilm phenotype compared to planktonic phenotype varies from 20 fold to 1000 fold higher [75]. The mechanisms for biofilm antibiotic resistance are complex and include: 1) slow penetration and failure to penetrate beyond the surface layers; 2) resistant highly protected phenotype state (a cell differentiation similar to spore formation); 3) and altered microenvironment with concentration gradients of nutrients and metabolic products existing across the layers or zones of biofilms [75]. Some antibiotics (ie aminoglycosides) with positive charge bind to negatively charged polymers in the biofilm and retard or slow the penetration [76].

## ADVANCES IN DIAGNOSIS OF IE

The diagnosis of IE is based on a combination of clinical, microbiologic and echocardiographic findings. The Duke criteria proposal in 1994 [77] and modified in 2000 [78], has been verified worldwide as a useful criteria for diagnosis of definite IE, with presence of two major criteria, or one major and three minor criteria. The emphasis of these diagnostic criteria relies heavily on positive multiple blood cultures with usual pathogens, echocardiographic evidence of vegetation or abscess, and predisposing cardiac valvular disease. The modified criteria no longer includes visible suspicious lesions for vegetations on echocardiography as a minor criteria, and Q-fever serology indicative of chronic infection has been included as a major criteria.

Echocardiography has been used as an important diagnostic tool in the evaluation of IE for over 25 years. Standard transthoracic echocardiography (TTE) was found to be insensitive to detect vegetations (around 50%), and transesophageal echocardiography (TEE) was shown to be significantly superior to detect vegetations in suspected IE more than a decade ago [79,80]. The vegetation size also affects the sensitivity of a TTE since only 25% of vegetations <5mm and 70% of those between 6-10mm are identified [80]. More recently, technological advances have resulted in improvements in TTE image quality, especially with harmonic imaging. In a relatively recent study 50 patients with native-valve IE were evaluated by both TTE and TEE examinations by high-end machines [81]. The sensitivity of TTE for vegetations was only 55% when using TEE as the “gold standard” where all patients had detectable vegetations by this latter technique. In previous studies using pathological examination for detection of vegetations the sensitivity of TEE was > 90%, whereas the sensitivity of TTE was only 58% [79]. The specificity of both imaging methods (with vegetation defined as a mass with independent motion attached to an endothelial surface) was excellent, 92% to 95%.

Patients with suspected IE by clinical criteria with a normal good quality TTE study are very unlikely to have IE, as the standard TTE have very high sensitivity in detecting abnormal valves which predispose to IE. 

Two other relatively small studies have reported their experience with different results using harmonic TTE for diagnostic evaluation in IE. In one study from Italy 139 patients underwent evaluation with harmonic TTE, TEE and TTE with fundamental imaging for the presence, dimension and characteristics of vegetations [82]. Thirty-five patients had definite IE. TEE detected vegetations in 33 patients (94%), harmonic TTE in 28 (80%), and fundamental imaging in 12 (34.2%). Compared to TEE fundamental TTE identified only one of seven abscesses (14% sensitivity), and harmonic TTE detected two of seven abscesses (28% sensitivity). A more recent smaller study of 36 consecutive patients, 19 had definite IE by TEE, found the harmonic TTE to have a sensitivity for detection of vegetations of 84%, specificity of 88%, positive predictive value of 89% and negative predictive value of 82% [83]. Hence, larger comparative studies are needed to give a final appraisal of the value of harmonic TTE in evaluating suspected IE. 

Based on current knowledge patients suspected of IE should be screened with TTE (harmonic), a negative study would make IE very unlikely for native valves. Equivocal results or prosthetic valves should undergo TEE, if the results are negative or not confirmatory then the TEE should be repeated in 7 – 10 days for those with high clinical suspicion of IE.

## ADVANCES IN MANAGEMENT OF IE

There have been no major advances in the management of IE in the past decade or more, although updates on guidelines have been recently published. Very few small randomized controlled trials have been published on the treatment of IE, and current guidelines by expert panels of various medical societies are based largely on observational data, in vitro studies, and animal experiments using rapidity and degree of bacterial killing on valves with abbreviated therapy rather than outcome.

Although diagnostic techniques, antibiotic choices and surgical techniques have improved over the past two decades, the complications and mortality of IE have not substantially changed. It is evident that we need to make earlier diagnosis, with specific microbial identification to institute early optimal therapy if we hope to improve the outcome. It is disappointing and surprising that even in a very recent survey only 71% of patients with IE had blood cultures taken before antibiotics [62]. Higher mortality and morbidity have been attributed to patients with blood culture negative IE, and up to 70% of this group are associated with prior antibiotics [84]. Thus, any patients with even the slightest suspicion of IE should have at least 3 sets of blood culture taken at different times (preferably at least an hour apart if the patient is not acutely ill or toxic). 

## ANTIBIOTIC THERAPY IN STREPTOCOCCAL IE

Oral streptococci of the viridans group are still common causes of community acquired native-valve IE or remote prosthetic-valve IE. Penicillin G intravenously alone for 4 weeks is still the best choice for fully susceptible strains, and the combination of intravenous penicillin combined with gentamicin or streptomycin for 2 weeks only is an alternative. This latter combination is mainly of benefit to shorten the duration of intravenous therapy, but poses greater risk for vestibular-ototoxicity and renal impairment. Thus patients should be given the option after discussion of the risk-benefit ratio. 

There have been emergence of oral streptococci with increasing resistance to penicillin worldwide, with relative penicillin resistance defined as minimum inhibitory concentration (M1C) > 0.12 ug/ml to ≤0.5ug/ml, and M1C>0.5 ug/ml are considered fully resistant. Updated treatment guidelines in 2005 [85] recommend a 4-week course of either penicillin or ceftriaxone in combination with gentamicin for the initial 2 weeks for native-valve IE with relative penicillin resistant streptococci based on expert opinion. However, there is paucity of data on this group of viridans streptococcal IE. In a recent report from the Mayo clinic 29 patients with IE secondary to penicillin-resistant viridans streptococci (with penicillin M1C ≤4.0 ug/ml), were identified over the past 38 years (^ 8 cases per decade) [86]. IE caused by these resistant strains accounted for only 6% of episodes of viridans streptococcal endocarditis with no significant increase over nearby four decades. Moreover, only 6.2% of the 29 cases (largest series reported) had streptococci that were highly resistant to penicillin (M1C > 2.0 ug/ml). In this series 19 patients had native-valve IE, and 9 of 10 patients were cured with 2.3 weeks of combined aminoglycoside and a penicillin, and after 1983 8 patients received an average of 5.1 weeks therapy, either penicillin or ceftriaxone plus aminoglycoside for the initial 2 weeks, or ceftriaxone monotherapy with cure in 7 patients. Nine of 10 patients with PVE were cured with 4.1 week regimen of either combination regimen or monotherapy with vancomycin or ceftriaxone. Thus, although current treatment guidelines should be effective, optimal therapy (least toxic and cost-effective) is unknown for penicillin resistant oral streptococcal IE. It is very likely that ceftriaxone alone would be just as successful as combination with an aminoglycoside but further trials are needed.

## STAPHYLOCOCCAL IE

*S. aureus* is the leading cause of health-care associated IE, early PVE and drug abuse-associated IE, and is associated with a high mortality and morbidity for left-sided involvement. Right-sided *S. aureus* IE in predominantly IVDA is a much more benign disease with better outcome and complications than left-sided *S.aureus* IE. The cure rates of right-sided IE are usually > 90%, whereas left-sided *S.aureus *IE cure rates are about 60 - 65 %. The difficulty in maintaining intravenous access and compliance with prolonged treatment regimens led to the adoption of shortened combination of beta-lactam resistant penicillin (naficillin, methicillin, cloxacillin etc) with gentamicin for 2 weeks in IVDA with right sided *S.aureus* IE in the late nineteen eighties. In a open study of 50 consecutive cases of methicillin-sensitive *S.aureus* (MSSA) right-sided IE in IVDA managed in San Francisco, 47 (94%) were cured with 2 weeks of intravenous nafcillin and tobramycin [87]. Subsequently many centers world-wide adopted this abbreviated regimen as standard therapy for uncomplicated right-sided *S.aureus *IE. However, in the mid-1990 an open randomized study from Spain, found that intravenous cloxacillin monotherapy for 2 weeks was just as effective as combined cloxacillin plus gentamicin for the same period, cure rates 34 of 38 (89%) versus 31 of 36 (86%), respectively [88]. Thus suggesting that either combined or monotherapy with a beta-lactamase resistant penicillin should be satisfactory for most cases of uncomplicated MSSA right-sided IE. It should be noted that the sample sizes of these reports are not large enough to adequately detect even a 20% difference in clinical response.

However, short-term therapy with vancomycin or teichoplanin in combination with gentamicin is not very effective for right-sided MSSA IE (60-70%) [89]. There is also recent retrospective data that IVDA with MSSA IE who received vancomycin had higher infection-related mortality than those treated initially with a beta-lactam agent in Detroit [90]. These results lend support to the impression that vancomycin is inferior to beta-lactam antibiotics for serious MSSA infections.

In patients with left-sided MSSA IE the treatment of choice should be a beta-lactam agent for 4-6 weeks. Although experts and textbooks recommended adding gentamicin for 3-5 days initially, there is no proof of significant clinical benefit. In a prospective mulitcenter study combining gentamicin with nafcillin for the initial 2 weeks did not improve outcome or morbidity, but the combination resulted in earlier cessation of bacteremia by 1.3 days over monotherapy [91]. Since there was greater toxicity with the gentamicin some experts and guidelines recommend gentamicin for only 3-5 days in fulminant cases of MSSA IE. A recent meta-analysis of comparative trials to assess the role of aminoglycoside in combination with β-lactam agent for treatment of IE has been reported [92]. Five comparative trials (four randomized) were analyzed, with 4 studies on *S.aureus *native-valve IE (N=261 patients). There was no significant differences between monotherapy and combination-therapy regarding mortality, treatment success, or relapse of IE. Nephrotoxicity was more common with the combination of a aminoglycoside than monotherapy, Odds Ratio (OR) 1.72, p=0.020 [92].

There is no clinical study addressing the issue of duration of antibiotic therapy in MSSA native-valve IE. As a general rule 4 weeks intravenous therapy should be adequate for uncomplicated cases, but 6 weeks therapy is preferable for complicated cases with perivalvular abscess.

## ENTEROCOCCAL IE

Traditionally it has been recommended to treat enterococcal IE with a combination of high dose penicillin G (24 million units/day) or ampicillin with gentamicin for 4-6 weeks. Vancomycin is an alternative (with gentamicin) for patients allergic to penicillin or for *Enterococcus faecium *resistant to penicillin/ampicillin. These recommendations were based on earlier case series showing high failure to monotherapy, and in vitro studies showing no single antibiotic was sufficiently bactericidal for *Enterococcus* species, and limited animal experiments. Combination with gentamicin was selected for strains inhibited by <500 ug/ml of gentamicin, as synergy with penicillin or vancomycin would be predictable. For gentamicin highly resistant strains (M1C ≥ 500 ug/ml) susceptibility should be done to streptomycin (which would produce synergistic killing with the penicillin or vancomycin if the organism was inhibited by < 1000 ug/ml).

A significant problem with this regimen was a high risk of gentamicin toxicity (25 – 40%) when administered for ≥4 weeks, especially since enterococcal IE occurred most often in older patients. A recent prospective observational study on the largest reported series of enterococcal IE have challenged the need for ≥ 4 week of aminoglycoside. A nationwide prospective study in Sweden between 1995-1999 identified 93 cases of enterococcal IE, 11% of 881 definite episode of IE [93]. Study patients received a median duration of aminoglycoside (in combination with penicillin/ampicillin or vancomycin) for 15 days and total antibiotic duration for 42 days. The overall cure rate was 81% with 16% mortality and 3% relapse, similar to results from previous studies with combination of 4-6 weeks [94]. Thus, the current best available data indicate that combination with aminoglycoside should be limited for the initial 2 weeks for treatment of enterococcal IE to obtain optimal results and reduce aminoglycoside toxicity.

Patients with enterococcal IE due to gentamicin and streptomycin high level resistance strains has been a therapeutic challenge. Occasional cases have been reported to be successfully treated with prolonged high dose ampicillin monotherapy and surgery, but no large series have been reported. Although enterococcal species are generally resistant to all cephalosporins there have been a few in vitro studies and limited animal experiments demonstrating synergy with ampicillin-ceftriaxone against enterococci [95]. In a small observational study from Spain 21 patients with high-level aminoglycoside resistant (HLAR) *E. faecalis* IE and 22 patients with non-HLAR *E.faecalis *were treated with 6 weeks of intravenous ampicillin (12g/day) plus ceftriaxone (4g/day) with cure rate of 67.4% (29 of 43 patients) [96]. Before this regimen be adopted as standard treatment for HLAR enterococcal IE, it would be preferable for an international multicenter trial be conducted to compare this combination to ampicillin alone. It should be noted the cure rate was only 52% (11 of 21 patients) in cases of HLAR enterococcal IE receiving the antibiotic combination without surgery [96].

## PROSTHETIC VALVE IE (PVE)

Recent international prospective observational study, involving 61 centers in 28 countries, has identified *S.aureus* (23%) as the most common cause of PVE, followed by CoNS (16.9%) from a cohort of 556 cases [97]. The majority (71%) occurred within the first year of valve implantation. Complications of PVE are common and include heart failure (32.8%), stroke (18.2%), intracardiac abscesses (29.7%), need for cardiac surgery (48.9%) and in hospital death (22.8%) [97], despite standard recommended combination therapy. Current guidelines for therapy of staphylococcal PVE are based on in vitro studies, animal models with soft tissue foreign-body related infections, and from retrospective data obtained in the early 1980s.

Current guidelines [85] recommend initial triple drug regimen (β lactam agent plus gentamicin plus rifamipin) for methicillin-sensitive strains of staphylococcal PVE; or vancomycin-gentamicin-rifampin for methicillin-resistant staphylococcal PVE for a total of 6 weeks. But there is little evidence to support these recommendations. In the 1980s Karchmer *et al. *[98] reported their experience on 75 episodes of CoNS PVE, 80% of which were methicillin resistant (MRSE). Vancomycin monotherapy was only effective 3 of 6 cases (50%), vancomycin plus rifampin in 7 of 8 (87.5%), vancomycin plus gentamicin in 5 of 5 (100%), and vancomycin-rifampin-gentamicin in 6 of 8 (85.7%) of cases. This data suggest that vancomycin monotherapy is inadequate and that vancomycin should be used in combination with either gentamicin or rifampin. The argument for triple therapy with gentamicin for initial 2 weeks is to reduce the risk of rifampin-resistant mutants. In a prospective randomized small treatment trial of MRSE PVE (published in abstract form only) no difference in cure rates (78%) was found between dual therapy (vancomycin-rifampin) versus triple therapy (vancomycin-gentamicin-rifampin), but rifampin resistance developed in 37% of the dual-therapy versus none in triple therapy group [99]. In an experimental rabbit model of native-valve IE with MRSE, 2 days of various therapies were analysed: monotherapies (vancomycin or gentamicin or rifampin), versus triple combination (vancomycin-gentamicin-rifampin) were compared to dual combination (vancomycin-gentamicin, or vancomycin-rifampin) [100]. Outcome measures analyzed were bacterial load of vegetations (colony forming units/gram [CFU/g]), and sterile vegetations 7 days after therapy. The mean log CFU/g vegetation was significantly higher for monotherapies 4.5-7.1 log CFU/g (with no sterilization) versus vancomycin-gentamicin 3.3 ± 1.3 log CFU/g (1/7 sterilization); vancomycin-rifampin 2.7 ± 1.2 log CFU/g (2/7 sterilization), triple combination 2.1 ± 0.2 (5/7 sterilization). The triple combination was significantly better for rapidity of sterilization of vegetations versus monotherapies or vancomycin-gentamicin (p<0.05), but was not significantly superior to vancomycin-rifampin dual combination [100]. Thus, if triple-therapy is being used for PVE it would be prudent to limit the duration of gentamicin for 5-7 days to reduce the risk to toxicity as there is no good clinical evidence that triple-therapy (vancomycin-gentamicin-rifampin) for both MRSA or MRSE infections is necessary.

There is even less data to support use of triple combination (nafcillin-gentamicin-rifampin) for MSSA PVE as recommended by guidelines [85]. Unlike MRSA strains of MSSA are readily killed by low concentrations of β-lactam agents. Thus, the use of rifampin as adjunctive therapy for severe MSSA infections is controversial. In a recent review of the adjunctive use of rifampin for the treatment of severe *S.aureus *infection it was concluded that the in vitro results of interactions between rifampin and other antibiotics are variable and method dependent and often do not correlate with *in vivo* findings [101]. Animal studies tended to show a microbiologic benefit of adjunctive rifampin use, particularly in osteomyelitis and infected foreign body soft tissue infection models. There has been no valid prosthetic valve IE model studied to date. However, clinical studies so far have failed to show a benefit of adjunctive therapy with rifampin [101], and further multicenter international trials are needed. 

In a recent retrospective cohort analysis of native-valve *S.aureus* IE 42 cases were treated with addition of rifampin (median 20 days) and 42 cases were not (controls) [102]. Cases treated with rifampin were more likely to have longer duration of bacteremia (5.2 *vs*. 2.1 days, p=<0.001) and were less likely to survive (79.9 *vs*. 95%, p=0.048) than controls. Unrecognized significant drug-drug interactions with rifampin occurred frequently (56%), and significant hepatic transaminase elevations occurred in 9 cases (21.4%), all of whom also had hepatitis C infection [102]. Hence adjunctive rifampin for even MSSA PVE is at best of questionable benefit, and potentially could be harmful. Currently there is no evidence that MSSA PVE should be treated any differently from MSSA native-valve IE. As noted earlier unlike foreign body soft tissue or bone infections, PVE can often be cured with medical therapy alone. Moreover, both native-valve IE and PVE epitomize biofilm infections, which are represented by the vegetations on the valves. There is valid argument (but controversial) that earlier surgical intervention should be performed for *S.aureus* PVE to improve outcome.

## SURGICAL THERAPY FOR IE

The indications and guidelines for management of IE have recently been updated [85]. *S.aureus* PVE is associated with high mortality and morbidity, including valve-ring abscess, dehiscence of the valve, embolization and severe heart failure. Early cardiac surgery has been advocated for these cases before attaining the usual indications for surgery to reduce these complications and high mortality. A recent retrospective review of *S.aureus* PVE from the Mayo clinic analyzed 55 cases to identify prognostic factors [103]. Twenty-three patients were treated medically and 32 patients had surgical intervention. Mortality was higher in the medically treated versus surgically treated patients (48% *vs.* 28%). Multivariate analysis showed that severity of illness (American Society of Anaethesiology (ASA) class IV) and bioprosthetic valves were independent predictors of mortality. Predictors of good outcome for medically treated patients included age < 50 years, ASA score III, and without cardiac, central nervous system or systemic complications [103]. The limitations of this study include retrospective collection of data and small sample size. Thus, the improved outcome of surgically managed patients could be from selection bias.

In the largest prospective observation cohort study of PVE ever reported (N=556), in hospital mortality for *S.aureus* PVE was still high (44/128 [34.4°]), despite use of recommended standard combination of antibiotics and early surgical intervention in many cases (48.9%) [97]. The strongest predictors of mortality were persistent bacteremia (>7 days), heart failure, intracardiac abscess, and stroke. Persistent bacteremia is independently associated with *S.aureus *IE. In a recent analysis of 61 *S.aureus *PVE from merged database of an international collaboration early valve replacement was not significantly associated with a survival benefit (overall mortality 47.5%) but patients who underwent early valve replacement for cardiac complications had the lowest mortality rate (28.6%) [104].

Since there has been no randomized controlled trial to assess the benefit of surgical therapy in general for IE, prospensity analyses have been used to control for bias in treatment assignment and prognostic imbalance to evaluate surgical intervention. Using prospensity analysis surgical therapy was significantly associated with lower mortality in patients with moderate to severe heart failure in left-sided native-valve IE (14% *vs*. 51%, p= 0.001) [105]. In a cohort of 367 patients with PVE using prospensity analysis in hospital mortality was similar for patients treated with surgery compared to medical therapy (25% *vs*. 23.4%, respectively) [106]. After adjustment for factors related to surgical intervention, brain embolism and *S.aureus* were independently associated with mortality, with a trend towards benefit with surgery (OR= 0.56, 95 CI 0.23 – 1.36). Timing of the survival end-points may have affected the outcomes, as in a previous small single center study of 66 patients with PVE, benefit of surgery was only evident in long term follow-up (survival at 10 years was 28% in the medically treated patients *vs*. 58% in those treated surgically) p=0.04) [107]. The debate over the need for early surgical intervention will remain unresolved until international collaboration result in a large randomized, prospective trial to answer this question.

## PACEMAKER AND CARDIOVERTER-DEFIBRILLATOR INFECTION

Permanent pacemakers (PPMS) and implantable cardioverter defibrillators (ICD) are increasingly being used for the management and prevention of serious cardiac rhythm disturbances. In the United States there was a 42% increase (from 3.26 per 1000 to 4.64 per 1000) in the cardiac device implantation rate among medicare beneficiaries from 1990 to 1999 [108]. The rate of PPM or ICD infection has varied from 0.13% to 19.9% but has increased by 123% over the past decade [108]. Several host-and procedure-related factors have been reported to increase the risk of infection with the devices, including diabetes mellitus, malignancy, operator inexperience, advanced age, corticosteroid use, anticoagulation, chronic renal failure, recent device manipulation and bacteremia from a distant focus of infection [109]. In a recent analysis of 29 PPM-related infections and 58 controls multivariate logistic regression model identified long-term corticosteroids use (OR, 13.90, p=0.03;and the use of > 2 pacing leads versus 2 leads (OR, 5.41, p=0.01) as independent risk factors for infection [110]. Preoperative antibiotic prophylaxis had a protective effect in reducing PPM infections (OR, 0.087, p= 0.005).

In a retrospective population-based cohort study from 1975 to 2004 in Minnesota, 1524 patients with cardiac devices were identified, with a total person-time follow of 7578 years [111]. The incidence of definite device infection was 1.9 per 1000 device years, with pocket infection without bacteremia in 1.37 per 1000 device years, and with pocket infection and bacteremia or device-related IE in 1.14 per 1000 device years. Of the 1524 patients from the full cohort, 1087 were included in the analysis with 6 pocket infections without bacteremia and 78 cases of blood stream infection. The most common organisms in patients with bacteremia were *S.aureus *(28%, of which 40% were MRSA), *Escherichia coli *(27%), *Klebsiella* species (9%), *Enterococcus* species (8%), α-haemolytic streptococci (8%), CoNS (6%), and others. Six (18%) of 33 who had echocardiogram had demonstrable vegetations (seen only on TEE), present on the lead only in 2 cases, the valves and lead in 2 cases, and valves only in another 2 patients. None of the episodes of gram-negative bacilli infection were definite cardiac device infection, and originated from the urinary tract or intraabdominal source [111]. Twelve (54.6%) of 22 cases of *S.aureus* bacteremia had definite or possible cardiac device infection *vs*. 3 (12.0%) of 25 cases of gram negative bacteria were possible (not proven) device infection (p=0.004). The accumulative probability of device infection was higher among those with a defibrillator compared to those with a pacemaker in this study (p < 0.001).

Other studies have reported that generator pocket infection is the commonest clinical presentation (up to 83%) with cardiac device related IE in only about 10% [110]. However, electrode lead IE occurs in less than 1% of PPM or ICD implants (0.58 and 0.65%, respectively, in a recent study) [112]. Superficial cellulitis of the wound (implantation site) usually occur within the first two weeks after surgery, usually no drainage is present and blood cultures are negative. The infection generally resolve with intravenous/oral antibiotics with a narrow spectrum β-lactam agent [113]. Pocket infection typically develops within the first 6 months of the pulse generator or battery-pack implantation, or device manipulation. Even though the infection may manifest itself several months after implantation, there is evidence the organisms (usually of low virulence, ie CoNS or diphtheroids) were inoculated at the time of surgery [114]. Infection with more virulent organisms such as *S.aureus* usually present much earlier after surgery. Pocket infection generally present with local findings (erythema, tenderness and /or drainage) and 20% may have constitutional symptoms (fever, chills) [114]. Late pocket infection after one year of implantation is often associated with recent surgical revision, erosion through the skin from inadequate fixation and occasionally from bacteremia of another source. Pocket infection, unlike superficial cellulitis, requires removal of the lead-generator plus specific antibiotics (depending on the organisms and susceptibility), and a new pacemaker/ICD in a remote location after resolution of the local wound infection (if still required as determined by repeat electrophysiological study).

Pocket infection is believed to be the most common factor leading to PPM/ICD endocarditis with the same organism (staphylococci mainly), however, up to 60% of patients have no focal signs of pocket infection [115]. Clinical presentation of these device related IE consist mainly of fever, occasionally with pulmonary infiltrates, and late onset electrode endocarditis can also result from hematogenous seeding of *S.aureus*, streptococci, enterococci and rarely coliforms. In a recent prospective study of 224 patients suspected of pacing system-related infections *S. epidermidis *accounted for 66.9%, other CoNS for 28.5%; 33 patients had IE with 30 demonstrating the same organisms on blood culture and lead culture [116]. Also 25% of those with endocarditis has multiple organisms (>1 strain or species of bacteria). For the diagnosis of pacemaker/ICD IE the sensitivity of TTE in detecting lead vegetations ranges from 22% to 30%, whereas TEE has a sensitivity of up to 95% [117].

Management of pacemaker/ICD related IE is with appropriate intravenous bactericidal agent depending on culture and susceptibility results for 4-6 weeks. Removal of the entire cardiac device is recommended as observational studies showed high failure rate and relapse with medical therapy alone. In a previous retrospective case series of 123 patients with pacemaker/ICD infection only 0.86% of 117 patients with removal of the entire device had infection relapse versus 3 of 6 (50%) without removal of the hardware (p=0.03) [118]. In a smaller prospective study of 31 patients the only prognostic factor for failure of treatment or mortality was the absence of surgical therapy (p<0.0001) [119]. Failure to remove infected PPM/ICD has been associated with almost 3-fold increased risk of dying (6.7% *vs*. 17.6%) [114]. In a previous review it was found that the mortality rate in patients with PPM/ICD IE treated with antibiotics alone range from 31% to 66% *vs*. 13% to 33% (mean 18%) in those treated with antibiotics combined with removal of the entire device [120].

In patients with PPM/ICD in place for many years the electrode can become embedded and fixated by fibrocollagenous tissue. Conservative methods for removal of the electrode now include “locking stylet” affixed close to the distal end of the electrode; telescoping sheath to disrupt fibrous attachment mechanically; and laser sheath to photoablate the fibrous attachment. These techniques result in lead extraction of 81% to 93%, but complications (including tamponade) can occur in up to 3.3% [121]. Patients with removal of the cardiac device should have re-evaluation to determine need for re-implantation as a significant proportion (13% to 50%) may no longer require pacing support [122]. Those requiring re-implantation of the device should be done at a separate site when the patient is afebrile and no longer bacteremic, and with resolution of any pocket-site infection.

Although antibiotic prophylaxis is commonly used for PPM/ICD implantation there is no large randomized trial to prove efficacy. In a meta-analysis of 7 randomized trials with 2023 patients (new implants or replacements) systemic antibiotic prophylaxis (semi-synthetic penicillin or cefazolin), appeared to have a consistent protective effect in preventing short-term pocket infection, skin erosion or bacteremia [123].

## INFECTION AND ATHEROSCLEROTIC HEART DISEASE

Up to recently (< 5 years) there was great interest and intensive research internationally on infectious agents playing a role in atherosclerosis and the complications, such as ischaemic heart disease and stroke. As a result of negative therapeutic trials showing no benefit of antimicrobials in recurrent heart attacks, strokes or death in relatively large, mulitcenter, randomized, controlled trials (RCTs) [124-127], there has been a dramatic decline in research and medical or public interest. Is this justified and have these RCTs disprove the theory that some infections can play a role in the pathogenesis of ischaemic heart disease? The resounding answer is no! 

The design of the RCTs and several other limitations among them leaves the issue unresolved. This is similar to negative therapeutic trials recently reported with agents specifically aimed to improve HDL cholesterol, despite the large body of data that shows that low HDL predispose to CAD and high HDL is cardioprotective [128, 129]. 

The recent large RCTs studied patients with known CAD, either chronic stable disease or after acute coronary syndrome to assess the value of antimicrobials (newer macrolides or quinolones) in preventing secondary cardiac events. The hypothesis generating these RCTs is that *Chlamydia pneumoniae *(a common respiratory pathogen) can precipitate acute cardiac events or play a role in destabilizing existing coronary plaques. The assumption of these trials is that persistent viable *C.pneumoniae *exist in coronary arteries in patients with significant anti-*C.pneumoniae *antibodies and in the majority of patients with CAD irrespective of antibodies [125].

Although the concept of infectious diseases as potentially playing a role in the pathogenesis of atherosclerotic disease has existed for over a century, renewed interest was stimulated by epidemiological studies showing association of CAD and antibodies to *C.pneumoniae *in the past 2 decades. The strongest association between ischaemic heart disease/stroke and infection exist primarily for *C.pneumoniae *and bacteria causing periodontitis (ie *Porphyromonas gingivalis*) [130]. However, the strength of the association between these infections and CAD by clinical and epidemiological studies have been mixed and inconsistent. Whereas numerous retrospective case-control and cross-sectional studies had shown significant association between past infection with *C.pneumoniae* and periodontal pathogens with CAD, prospective cohorts and meta-analyses of these studies have not confirmed significant correlation or association with infection and atherosclerotic heart disease or stroke [131,132].

Atherosclerosis the undergoing cause of CAD and stroke, is an inflammatory (low grade) disease with pathobiologic mechanisms which are common to the host’s responses to many chronic infectious diseases. Microbes may potentially play a role in the development of CAD at several steps: i) initial endothelial vascular injury via direct or indirect mechanisms that could initiate atherosclerosis; ii) acceleration of early atherosclerosis via local increase in LDL and oxidized LDL by cytokines stimulation or by predisposing to abnormal lipid profile; iii) precipitation of acute events by predisposing to vulnerable plaque or activation of the coagulation cascade [131].

The biologic mechanisms by which these infections could play a role in atherogenesis are fairly well established by in vitro, ex vivo and in vivo (animal models) [131, 132], summarized in Table **2**. Moreover, numerous studies have found evidence of *C.pneumoniae* in arterial plaques by several different techniques (immune histochemistry, in situ hybridization, PCR, electron microscopy and cultures) but rarely in normal arteries [131]. The mean prevalence of *C.pneumoniae *in atherosclerotic plaques is about 40 -50%, and the presence of the organisms in CAD or other arteries do not correlate with the standard antibodies measured in clinical trials [131, 133]. Hence recent RCTs that determine inclusion of patients by anti-chlamydial antibodies or the assumption that most patients have chronic persistent *C.pneumoniae* infection are likely to be under-powered.

Animal experiments in rabbits have demonstrated that *C.pneumoniae *can initiate early atherosclerotic changes in the aorta without hyperlipidemia [134]. However, the majority of animal experiments have demonstrated that *C.pneumoniae* [131] and *P.gingivalis* can accelerate atherosclerosis in the presence of hyperlipidemia [135]. Although theoretically these microbes could lead to precipitation of acute cardiac events by destabilization of plaques through induction of metalloproteinases or gelatinase by macrophages [136,137]; or by inducing tissue factor to cause or precipitate acute thrombosis (clot) [138], no animal models have shown this effect. Thus, the recent RCTs were designed to prevent secondary cardiac events (or precipitation by chronic *C.pneumoniae* infection) which have never been demonstrated to occur in animal models.

Moreover, although Muhlestein *et al. *[139] showed in the rabbit model (fed cholesterol enriched diet) that azithromycin (a newer macrolide) could prevent acceleration or enhancement of atherosclerosis (when treated immediately after infection), delayed treatment has been shown to be ineffective in preventing artherosclerotic changes [140]. Furthermore, there is evidence that single agents (such as azithromycin or ofloxacin) cannot eradicate *C.pneumoniae* in a chronic persistent state in either a continuous cell culture model or experimental murine pneumonitis model [141, 142]. Hence the antibiotics used in the recent RCTs [124-127] are not very effective in eradicating *C.pneumoniae *from tissues in animals or even within human monocytes [143]. Thus the negative results from these RCTs could be secondary to an ineffective regimen. Currently the best choice of agents to eradicate persistent chronic infection with *C.pneumoniae *is unknown but may be a combination with rifampin [142].

Other factors that could affect the outcome of these negative RCTs include the fact that the majority of patients were already receiving optimal therapy, such as selective β-blocker, anti-platelet drugs (ie aspirin) and “statins”. Thus making it extremely difficult for any additional therapy to show any difference above standard treatment. This also applies to lack of benefit seen with HDL-modifying agents. Moreover it has been demonstrated in vitro that “statins” can inhibit *C.pneumoniae* in cell culture and suppress the inflammatory or cytokine response to this microbe that may be playing a role in atherogenesis [144]. Recent studies demonstrate that simvastatin reduces *C.pneumoniae*-mediated histone modification and gene expression in cultured endothelial cells, thus down regulating cytokine production that are important in initiating and accelerating atherosclerosis [145]. Furthermore in acute infection of swine with *C.pneumoniae* via the respiratory tract, there is impairment of the muscarinic and kinin-related reactivity of coronary circulation, which can be prevented by simvastatin [146].

## RECENT ADVANCES IN INFECTIONS AND ATHEROSCLEROSIS

There has been no significant advances in the last 5 years to further establish the relationship between *C.pneumoniae* or *P.gingivalis* with CAD/stroke in human trials. To further establish this relationship would need more specific and sensitive diagnostic blood tests that reflects chronic persistent infection and correlates well with the presence of *C.pneumoniae* in atheromatous plaques. One potential candidate for diagnosis of persistent *C.pneumoniae* infection is a recently developed enzyme immunoassay [EIA] for quantification of chlamydial LPS in serum [147]. However, combined serological and pathological studies need to be performed to confirm the correlation of the cLPS and presence of the organisms in atheromatous plaques. Another potential candidate diagnostic test is determination of chlamydial heat shock protein (cHSP) antibody [148]. However, only one small study to date has been performed to determine correlation with pathological evidence of *C.pneumoniae* in atheromas [133]. Commercialization of Hsp- 60 monoclonal antibody (Genway Biotech) has now paved the way for further larger histopathogical-serological studies by other centers. This is crucial before undertaking any further large prospective epidemiological cohort studies or RCTs of any therapeutic agent.

A key issue in the hypothesis of *C.pneumoniae* role in atherogenesis is the ability of this microbe to persist intracellularly as an aberrant body, which has been mainly described in chronic *Chlamydia trachomatis* infections [149]. These persistent aberrant forms are in an arrested state in development, non-culturable, and explains the low yield of culturing human atheromas for *C.pneumoniae* [131]. In a recent study the presence of aberrant or persistent bodies of *C.pneumoniae *were demonstrated in human coronary atheromas by immuno-gold electron microscopy technique [150]. To define the serological responses to *C.pneumoniae *antigens that are associated with persistent infection, another recent report described antibody patterns of sera from subjects with and without evidence for persistent *C.pneumoniae *(determined by multiple PCR analysis at different times of peripheral blood mononuclear cells and vasculatory samples) by using proteomics, combined with 2-D gel immunoblotting [151]. In this study a unique antibody response pattern (by differential reactivity for 12 proteins) were found which reflected persistent *C.pneumoniae, *and was not predicted by the current gold standard for sero-diagnosis (the immunofluorescence test). The method used in this report, however, would be too expensive, time consuming and cumbersome to used for large multicenter population studies and clinical trials. Thus application of this data to produce a simpler, easily performed test would be desirable for future investigation.

As mentioned previously *C.pneumoniae* theoretically could precipitate acute vascular events by upregulation of matrix metalloproteinases (MMP) in coronary atheromas leading to vulnerable plaques. A recent study in human coronary plaque specimens (N=31) demonstrated significant association between expression of MMP-9 and the intravascular presence of *C.pneumoniae* [152]. There is also experimental evidence in mice that apoptosis of vascular smooth cells (VSMC) is responsible for thinning of fibrous cap, loss of collagen and matrix with intense inflammation, leading to plaque vulnerability (features that predispose to plaque rupture, thrombosis and acute ischaemic events) [153]. *C.pneumoniae *has recently been shown to induce apoptosis and necrosis of human coronary artery endothelial cells [154], but the organism can also infect human VSMC cells [131].

There is no simple blood test to assess periodontal disease and dental examination is necessary. There is recent data that have shown a direct relationship between periodontal microbial burden and subclinical atherosclerosis in 657 subjects by measuring mean carotid artery intima-media thickness [155]. In a recent cross-sectional study of 3585 participants ≥ 40 years old in the National health and Nutrition Examination Survey (NHANES) during 1999-2002, a significant association was found between periodontal disease and peripheral vascular disease (OR=2.25, p<0.05), which remained significant after adjustment for other factors [156]. Over the past decade or more there have been numerous basic research studies that demonstrated that protease-activated receptors are important in the pathobiology of hemostasis, thrombosis and vascular disease (all key elements in atherogenesis) [157]. *P.gingivalis* has recently been shown to activate protease-activated receptor -2 in the murine model of infection [158], providing further biological support for its possible role in atherogenesis.

In conclusion the association of infections, particularly *C.pneumoniae* and periodontal disease, with atherosclerosis and the complications still is a viable hypothesis with good evidence supported by fundamental research.

## FUTURE DIRECTIONS

Progress in the understanding of infectious diseases affecting the heart has been significant over the past decade. Large randomized therapeutic trials, however, have been lacking due to the low prevalence and incidence of these diseases. Recent international collaboration of several reknown centers have reported large perspective observational data on IE, but it is time for these groups to design and perform meaningful RCTs. For the 21^st^ Century we need to derive guidelines based on large randomized trials in IE, myocarditis and others rather than on substandard observational data.

With respect to proving causality between ischemic heart disease/stroke and infections, this will be a daunting task for a disease with multiple etiologic conditions. Large randomized controlled trials should not be performed until several prerequisites are fulfilled (re: *C.pneumoniae*): 1) an easily available test that can be applied to large populations that is predictive of persistent, chronic infection in atheromas; and 2) a proven effective regimen in suitable animal models. Finally these therapeutic RCTs should not be performed on subjects with established CAD already on standard regimens that include statins. Trials should be designed in middle-aged population with subclincial disease on imaging, not on other treatment, and to follow the progress of these atheromas with and without specific therapy for *C.pneumoniae.* It will be difficult to design a proper randomized trial for people with periodontal disease to offer proper dental care versus no care, as this would be unethical. Whether standard dental care versus intensive periodontal care is a feasible alternative, depends on previous trials showing that intensive periodontal care results in better periodontal health and retention of teeth in the long term. 

## Figures and Tables

**Table 1:** Biologic Mechanisms of Infections on the Heart

Disease	Direct Invasion	Indirect Effect	Autoimmune
Rheumatic carditis (Group A streptococcus)	-	+ (molecular mimicry)	+++
Pericarditis	+	-	-
Viral Myocarditis	+	-	++
Chagas disease	+ (early, persistent)		++ (late)
Diphtheria	-	+ (toxin)	-
Toxic myocarditis (Sepsis, toxic shock syndrome)	-	+ (Excessive inflammatory cytokines, catecholamines)	-
Bacterial/fungal myocarditis	+ (focal)	-	-
Infective endocarditis	+ (biofilm)	-	-
ICD/Pacemaker infection	+ (biofilm)	-	-

**Table 2:** Pathogenic Mechanisms of Infections on Atherosclerotic Heart Disease

	*Chlamydia Pneumoniae*	Periodontal Disease
Initiation	Endothelial invasion, HSP 60-related dysfunction	Indirect endothelial dysfunction by LPS, cytokines.
Early disease	Induce foam cells, oxidize LDL in macrophages; induce fatty streaks in rabbits	Induce foam cells, ↑CRP.
Acceleration	Enhance lipid-induced atheroma: ↑foam cells, ↑lymphocytes, SMC proliferation, ↑cytokines, activate NF-kB.	Enhance lipid-induced atheroma in rodents; upregulate activation of NF-kB
Precipitation	↑ MMP, gelatinase; ↑apoptosis, necrosis; ↑tissue factor, ↑PAI-1	↑inflammtion, procoagulation *via* proteinase activator receptor, activiate factor X.

Data derived from references [131, 132]. Abbreviations: Hsp= heat shock protein; LDL = low density lipoprotein; Lps= lipopolysaccharide; CRP= C-reactive protein; SMC= smooth muscle cell; MMP= matrix metalloprotease; NF-KB= nuclear factor kappa B; PAI-1 – plasminogen activator inhibitror-1
